# Nerve Wounds

**Published:** 1918-09

**Authors:** 


***Nerve Wounds.*** By J. Tinel. Trans. from the French by Fred. Rothwell. Lond. Baillière, Tindall, and Cox, 1917. Price 15 s. net. French version, *Les Blessures des Nerfs.* Paris, Masson et Cie, 1916.

This book is based upon a careful study of nerve injuries as seen during the war. In attempting to reproduce the main thoughts of the writer in abbreviated form, it is impossible to give a smoothly flowing account of all the observations included in this admirable and extensive work. Among the numerous points made by the author, the following stand out most prominently.

Examination of paralyzed nerves should not be made until several weeks after the wound and then repeated every few weeks so as to study the progress in the evolution of symptoms. There is never any inconvenience because surgical intervention is postponed for two to three months after the injury. In the early weeks inhibition may simulate destruction. The paralysis of anesthesia is often more extensive than would be associated with the real lesion. Again muscular atrophy and hypotonia require several weeks to develop while pains due to irritation frequently appear only after eight to ten days and sometimes increase for a month. Formication due to nerve pressure appears about four to six weeks after the wound and reaction or degeneration only after three to four weeks following the injury. In all suspected cases faradic contractility excludes real paralysis except cerebral palsies. Every paralysed muscle is painless to pressure and this total insensibility is a clear sign of absolute nerve interruption — on the other hand pain of muscle bellies under pressure is the best sign of nerve irritation. Idiomuscular reflex is always intensified in peripheral nerve lesions despite hypo or even complete atonia. Trophic disturbances are absent or slight in almost all nerve interruptions or simple compressions, whereas they are almost constant in nerve irritations.

The trophic disturbances described by Tinel are as follows :

1. Glassy skin which is dry, dull, and immobile.

2. Excessive sweating.

3. Desquamation in the area of the nerve.

4. Vasomotor changes, for example, cyanosis and redness and at times edema, — which may correspond more exactly to the nerve distribution than the sensory disturbances.

5. Ulceration is very rare and usually due to burn or pressure.

6. Temperature changes. Local rise in temperature present in slight nerve irritations.

7. Changes in skin appendages; hypertrichosis is almost constant.

8. Changes in the nails (grooving, splitting, curving, striation or atrophy) are common in serious nerve irritation.

9. Changes in bones articulations and aponeuroses and tendons with shortening of ligaments and muscles with possible loss of mobility due to ankylosis are not uncommon.

*Symptoms of nerve interruption following section, very severe compression or bruising* :

1. Immediate absolute paralysis.

2. Rapid disappearance of muscle tone.

3. Progressive muscle atrophy.

4. Reaction of degeneration beginning in two to three weeks.

5. Sensory paralysis.

6. Loss of muscle pain sense.

7. Pressure on nerve below lesion produces no pain or formication.

8. Absence of serious trophic changes.

*Symptoms of nerve compression with loss of conductivity for the time being* :

1. Paralysis usually as complete as that caused by nerve interruption.

2. More rapid but less intense muscular atrophy.

3. Relative preservation of muscular tone which is one of the best signs of simple compression.

4. Partial reaction of regeneration which is slow in evolution and is always incomplete.

5. Anesthesia variable in extent and degree.

6. Absence of pains at the level of lesion, in the course of the nerve or on pressure on the muscle.

7. Absence of formication (but there may be slight formication under pressure).

8. Absence of trophic disturbance is even more marked than in complete section.

*Note* : Only after a few weeks' observation can one judge of the necessity for surgical liberation. Simple liberation is usually sufficient. Resection would only be required if constriction has transformed the nerves into a fibrous strand.

*Symptoms of Nerve Irritation* :

1. Pain spontaneous in character; burning and pricking is characteristic.

2. The skin is hyperesthetic; at times, however, there is anesthesia of the skin with pain on pressure of the deep tissues. Frequently there is painful hyperesthesia which may exist with tactical and thermal hypoesthesia.

3. Trophic changes. In pronounced neuritic types, trophic changes are serious and while paralysis heals spontaneously, or after nerve liberation, pains however acute they may be, inevitably diminish whereas the trophic changes are obstinate, leading to fibrous changes which are hard to correct.

During the period of irritation the nerve is painful. Regeneration is slow and in obstinate cases liberation of the nerve gives variable results. At times resection is the proper procedure. In addition to the above severe neuritic type, there is, according to Tinel, what he calls an attenuated neuritic type and also a simple neuralgic type. The attenuated neuritic type is characterized by slight pain on pressure on muscle bellies, slight fibrous infiltration of muscle; a few aponeurotic or tendon contractions; slight cutaneous sclerosis with adherant integument. At times these contractions come on late, and the nerve injury having been associated with so few symptoms and so little irritation, it escapes observation. The simple neuralgic type follows slight bruises of nerve trunks often accompanied by more or less pronounced neuralgic symptoms. There are no paralyses, no trophic changes, only slight degree of weakness and no appreciable change in reaction. There is slight hyperesthesia to pin pricks instead of anesthesia. Opposed to this rather mild condition is the intense neuralgic type with burning pain called by Weir Mitchell and studied during the Civil War — Causalgia. This is found especially in the median and sciatic nerve. The pains come on usually four to five days after the wound. They reach their maximum in three to four weeks and then last months, gradually diminishing. The pain is severe, comes in paroxysms at the slightest touch or motion, though firm pressure may not cause pain. There is loss of sleep and appetite, the patient gets very irritable and unstrung. To protect their hands and feet they keep their hands moist or pour water into their boots. Vasomotor disturbances are evidenced by redness and dryness with exaggerated sweating of the skin, and the hyperesthesia extends far beyond the cutaneous areas of the affected nerves, suggesting an irritation of vascular nerves or of the sympathetic periarterial plexuses. These cases are particularly obstinate and hard to treat.

*Regeneration of nerves* is progressive and scarcely ever begins before four to six weeks after nerve suture. Regeneration shows itself by the following complex symptoms :

1. As the axones develop there is progressive formication.
2. There is gradual return of muscle tone and muscle sensibility.
3. There is return of electrical normal contractility.
4. There is return of sensation first protopathic, then epicritic, with return of deep sensibility preceding the others.
5. Return of motion.

*Prognosis* : 60 o/o to 70 o/o of nerve injuries regenerate spontaneously; about 10 to 20 o/o of these would have improved more rapidly by liberation of the nerve or resection and suture. 30 to 40 o/o require operation. Nerve suture done under favorable conditions almost invariably succeeds. Of 108 cases of suture or graft there are only 14 failures (operator, Professor Delagenière); early sutures are followed by more rapid regeneration but success has been seen 15 months after original injury.

*Indications for Operation* : It is well to delay two to four months except in simple compression or severe neuritis in view of the fact that spontaneous cures take place and operation should not be undertaken if there are signs of regeneration. The operative procedures recommended are the following :

1. Liberation of compressed nerve. This liberation must be complete and if the nerve has been changed into fibrous tissue it must be resected and the ends sutured together without tension.

2. Resection of nerve. The two freshened ends of the nerve are brought together with catgut neurilemma sutures. If a small space 1 to 8 mm. is left between the ends of the nerve this can be spanned by neuroglia cells. Union, if the two ends are approximated, begins on the fourth day.

3. Occasionally it may be necessary to introduce a graft between two nerve ends that cannot be approximated.

4. Incision into nerve sheath at level of interstitial hematoma and evacuation of the irritating clot at times is very effective.

5. It has been suggested that in the neuritic types alcohol injections be made into the exposed nerves.

9. It has been recommended in the causalgia cases to denude the arteries and veins of their perivascular sympathetic plexuses by circumcision of the vessels.

7. In addition to the operative treatment, the importance of protheses, diathermy, radiotherapy, electrotherapy and mechanotherapy should not be overlooked.

